# The Protective Effects of Levo-Tetrahydropalmatine on ConA-Induced Liver Injury Are via TRAF6/JNK Signaling

**DOI:** 10.1155/2018/4032484

**Published:** 2018-12-04

**Authors:** Qiang Yu, Tong Liu, Sainan Li, Jiao Feng, Liwei Wu, Wenwen Wang, Kan Chen, Yujing Xia, Peiqin Niu, Ling Xu, Fan Wang, Weiqi Dai, Yingqun Zhou, Chuanyong Guo

**Affiliations:** ^1^Department of Gastroenterology, Shanghai Tenth People's Hospital, Tongji University School of Medicine, Shanghai 200072, China; ^2^Shanghai Tenth Hospital, School of Clinical Medicine of Nanjing Medical University, Shanghai 200072, China; ^3^Department of Gastroenterology, Shanghai Tenth People's Hospital Chongming Branch, Tongji University School of Medicine, Shanghai 202157, China; ^4^Department of Gastroenterology, Shanghai Tongren Hospital, Shanghai Jiaotong University School of Medicine, Shanghai 200336, China; ^5^Department of Oncology, Shanghai General Hospital, Shanghai Jiaotong University School of Medicine, Shanghai 200080, China; ^6^Department of Gastroenterology, Zhongshan Hospital of Fudan University, Shanghai 200032, China; ^7^Shanghai Institute of Liver Diseases, Zhongshan Hospital of Fudan University, Shanghai 200032, China

## Abstract

**Aims:**

Levo-tetrahydropalmatine (L-THP) is an active ingredient of *Corydalis yanhusuo* W. T. Wang, which has many bioactive properties. Herein, we investigated the protective effects of L-THP on concanavalin A- (ConA-) induced hepatitis in mice and explored its possible mechanisms of these effects.

**Main Methods:**

Balb/c mice were intravenously injected with 25 mg/kg ConA to generate a model of acute autoimmune hepatitis, and L-THP (20 or 40 mg/kg) was administered orally once daily for 5 d before the ConA injection. The liver enzyme levels, proinflammatory cytokine levels, and other marker protein levels were determined 2, 8, and 24 h after ConA injection.

**Results:**

L-THP could decrease serum liver enzymes and pathological damage by reducing the release of inflammatory factors like IL-6 and TNF-*α*. The results of Western Blot and PCR indicated that L-THP could ameliorate liver cell apoptosis and autophagy. L-THP could suppress T lymphocyte proliferation and the production of TNF-*α* and IL-6 induced by ConA in a dose-dependent manner *in vitro*. Additionally, the protective functions of L-THP depended on downregulating TRAF6/JNK signaling*. Conclusion*. The present study indicated that L-THP attenuated acute liver injury in ConA-induced autoimmune hepatitis by inhibiting apoptosis and autophagy via the TRAF6/JNK pathway.

## 1. Introduction

The liver is the largest digestive organ and plays an important role in detoxification, metabolism, and immunity. Hepatitis is a disease characterized by the presence of alanine aminotransferase (ALT) and aspartate aminotransferase (AST) in the serum and hepatocyte apoptosis and necrosis in histology. Autoimmune hepatitis (AIH) is regarded as a chronic liver disease which has many causes [1]. While AIH is still considered a rare disease, its incidence rate has risen worldwide in recent years [2], becoming a global health burden. The administration of glucocorticoid combined with azathioprine has been used for many years in the clinic to control AIH, but the severe side effects of this treatment can be harmful. Therefore, animal models of AIH should be generated to facilitate research into the pathogenesis of AIH and to develop new targeted therapies [3–5].

An ideal model to investigate the mechanisms and treatments of AIH is essential for biological studies. The concanavalin A- (ConA-) induced AIH model is dose-dependent and simple to establish. ConA is a blood lectin extracted from the seed of *Canavalia ensiformis*. In 1992, Tiegs et al. successfully used ConA to establish an AIH mouse model [6]. The ConA injection could activate T lymphocytes, causing immune hepatitis and the release of inflammatory cytokines, including TNF-*α*, IL-6, IL-1*β*, and IFN-*γ*. Research has shown that high cytokine levels are closely related to the early liver injury [7].

Our previous studies showed that ConA injection resulted in increased levels of serum transaminases, as well as hepatocyte apoptosis and autophagy [8, 9]. Apoptosis and autophagy are two forms of programmed cell death. Apoptosis, which could be activated by a variety of factors, is used to eliminate abnormal or dead cells and maintain tissue homeostasis [10]. The morbidity and mortality of liver disease could be reduced by preventing apoptosis. Autophagy is an evolutionarily conserved pathway that is essential to many biological processes including inflammatory responses to promote cell survival [11, 12]; however, when overactivated, autophagy can lead to cell death [13]. Therefore, autophagy is considered a double-edged sword.

*Corydalis yanhusuo*, also called Rhizoma corydalis, is a traditional Chinese medicine. Levo-tetrahydropalmatine (L-THP), one of the major active components of *C. yanhusuo*, has been used as an anxiolytic and sedative drug in China for many years [14]. L-THP also shows potential for treating morphine [15], cocaine [16], and alcohol [17] abuse. Zhang et al. [18] found that L-THP could attenuate pain associated with bone cancer by inhibiting microglial cell activation and reducing proinflammatory cytokine TNF-*α* in the spinal cord. Moreover, L-THP has cardioprotective effects in myocardial ischemia-reperfusion injury by decreasing the accumulation of inflammatory factors such as TNF-*α* and the extent of apoptosis [19]. Furthermore, Min et al. [20] found that dl-tetrahydropalmatine (dl-THP), another major active component of *C. yanhusuo*, could exert inhibitory effects on liver injury induced by CCl4 in mice by reducing lipid peroxidation product. However, the function of L-THP pretreatment in ConA-induced AIH remains unclear.

This study explored the functions of L-THP in ConA-induced AIH and its underlying mechanisms of action. We showed that L-THP ameliorated ConA-induced AIH by modulating inflammation, apoptosis, and autophagy and that these activities were linked to the TRAF6/JNK pathway.

## 2. Materials and Methods

### 2.1. Reagents

Dimethyl sulfoxide (DMSO), L-THP, and ConA were purchased from Sigma-Aldrich (St. Louis, MO, USA). ALT and AST microplate test kits were purchased from Jiancheng Bioengineering Institute (Nanjing, China). Enzyme-linked immunosorbent assay (ELISA) kits for TNF-*α* and IL-6 were purchased from Anogen-Yes Biotech (Mississauga, Canada). The antibodies used in the study were from Cell Signaling Technology (Danvers, MA, USA) and included anti-IL-6, -LC3, -p-JNK, -TNF-*α*, -*β*-actin, -Bax, -Bcl-2, -cleaved caspase 3, -cleaved caspase 9, -Beclin-1, -TRAF6, and -JNK from Proteintech (Chicago, IL, USA). PCR kits were purchased from Takara (Dalian, China). RPMI-1640 medium was purchased from Thermo (Shanghai, China). The Pan T cell isolation kit was purchased from Miltenyi Biotec (Bergisch Gladbach, Germany).

### 2.2. Animals and Treatment

Male Balb/c mice (6–8 weeks old, 23 ± 2 g) were purchased from Shanghai Laboratory Animal Co. Ltd. (Shanghai, China) and were housed in a clean room at 24°C ± 2°C under a 12-hour alternating light and dark cycle, with free access to drinking water and food. All experiments were designed based on the National Institutes of Health Guidelines and approved by the Animal Care and Use Committee of Shanghai Tongji University (China). In the present study, we made every effort to minimize the pain of the mice.

ConA (5 mg/ml) was dissolved in saline solution, and the solution was then injected via the tail vein at a dose of 25 mg/kg to induce acute hepatitis according to a previously published protocol [8]. L-THP was dissolved in 1% DMSO, and the solution (at a dose of 20 or 40 mg/kg) was administered orally once daily for 5 d before inducing hepatitis. The 108 mice were randomly divided into six groups (*n* = 18 per group):

1. Normal control group: no treatment
2. DMSO group: mice were administered with 1% DMSO orally once daily for 5 d
3. L-THP group: mice were administered with 40 mg/kg L-THP orally once daily for 5 d without ConA injection
4. ConA group: mice were administered with 25 mg/kg ConA via the tail vein
5. Low L-THP + ConA group: mice were administered with 20 mg/kg L-THP orally for 5 d before a 25 mg/kg ConA challenge
6. High L-THP + ConA group: mice were administered with 40 mg/kg L-THP orally for 5 d before a 25 mg/kg ConA challenge

The normal control, DMSO, and L-THP groups were sacrificed after 5 d; in the other groups, six mice were randomly selected for sacrifice at the time points 2, 8, and 24 hours.

### 2.3. Biochemical Assays

After being stored at 4°C for 4–5 h, serum was isolated by centrifugation at 2000 ×g at 4°C for 10 min. Serum AST and ALT were determined using an Olympus AU1000 automated chemical analyzer (Tokyo, Japan). ELISA kits were used to detect the serum inflammatory cytokines. TNF-*α* and IL-6 levels were measured according to the manufacturer's instructions. To measure TNF-*α* levels, 50 *μ*l of Assay Diluent was added to each well of a 96-well plate at room temperature. Then we added 50 *μ*l of the serum sample to each well, covered the plate with a plate sealer, and incubated the samples at room temperature for 2 hours. Each well was then aspirated and washed for 5 times. 100 *μ*l of Conjugate was added to each well, and then we covered the plate with a new plate sealer and incubated the samples at room temperature for 2 hours. Then we aspirated and washed each well 5 times. In the dark conditions, we added 100 *μ*l Substrate Solution to each well and incubated the samples at room temperature for 30 minutes. After 100 *μ*l of Stop Solution was added to each well, a microplate reader set to 450 nm was used to determine the optical density of each well immediately. Similarly, the IL-6 levels were detected by a microplate reader set to 450 nm after a series of incubation, aspiration, washing, and the administration of Stop Solution.

### 2.4. Histopathology

After mice were sacrificed, half of the largest liver lobe was cut and fixed in 4% paraformaldehyde for at least 24 h. Then the sections were cut at a thickness of 3 *μ*m. And then the 3 *μ*m sections were stained with hematoxylin and eosin (H&E) staining. Pathological damage was observed by light microscopy, and the slides were blind to the observer.

### 2.5. Immunohistochemistry

After heating for 20 min at 60°C, the prepared 3 *μ*m paraffin sections were dewaxed with xylene and dehydrated in alcohol. After an antigen retrieval technique, the sections were incubated in bovine serum albumin (3%) for 20 min at 37°C to block endogenous peroxidase activity. Then the sections were incubated overnight using primary antibodies (1 : 500) including anti-TNF-*α*, anti-IL-6, anti-Bax, anti-Bcl-2, anti-Beclin-1, anti-LC3, and anti-TRAF6. On the second day, the sections were incubated using a secondary antibody. Then a diaminobenzidine kit was used to analyze the positive points. Finally, the liver sections were observed by light microscopy.

### 2.6. Western Blot Analysis

Liver tissues were stored at −80°C. Liver tissues were lysed using a radioimmunoprecipitation assay lysis buffer with protease inhibitors. A bicinchoninic acid protein assay was used to detect the protein concentration. Then the samples were boiled in 5x SDS-PAGE sample loading buffer. The proteins were isolated using 7.5%–12.5% SDS-polyacrylamide gels. Then the proteins were transferred to polyvinylidene difluoride (PVDF) membranes. Then the membranes were blocked in 5% nonfat milk (dissolved in PBS) for 1 h and were incubated with primary antibodies at 4°C overnight. On the next day, membranes were washed with PBST three times and incubated with secondary antibodies for 1 h at room temperature. Finally, we used the Odyssey imaging system to scan the membranes to detect the levels of specific proteins.

### 2.7. Reverse Transcription PCR (RT-PCR) and Quantitative Real-Time PCR (qPCR)

We extracted total RNA from liver tissues using the TRIzol reagent (Takara, Shiga, Japan) and transcribed into complementary DNA (cDNA) using the RT kit. Then we performed SYBR Green Quantitative RT-PCR to detect and analyze the expression of target genes using a 7900HT fast real-time PCR system (Applied Biosystems, New York, NY, USA). The primers used for qPCR are listed in Table 1.

### 2.8. T Cell Purification

Male Balb/c mice (6–8 weeks old, 23 ± 2 g) were sacrificed, and their spleens were collected. Under aseptic conditions, the cell suspension was isolated through a sterile plastic strainer (aperture: 0.0750 mm). After centrifugation at 100g for 5 min, the erythrocytes in the cell suspension were lysed with a red blood cell lysis buffer. Then the cells were washed twice with RPMI-1640 medium. Then the cells were suspended in complete RPMI-1640 medium. Trypan blue exclusion was used to determine the number of living cells, and viability of the splenocytes is more than 95% in all cases. Then, the Pan T cell isolation kit was used to purify T cells from splenocytes according to the manufacturer's protocols [21]. In brief, in MACS buffer (PBS, 0.5% BSA and 2 mM EDTA), a single-cell suspension was generated from the collected splenocytes above. Finally, T cells were selected after the cells were incubated with antibody cocktail and magnetic beads using a magnetic column. And the purity of CD3 T cells was more than 95%.

### 2.9. T Cell Proliferation Assay

The T cell density was adjusted for 2 × 10^6^/ml, and then the T cells were divided into the four groups: normal control group (no treatment), ConA group (ConA + T cell), 15 *μ*Μ L-THP group (ConA + T cell + L-THP 15 *μ*Μ), and 30 *μ*Μ L-THP group (ConA + T cell + L-THP 30 *μ*Μ). Except for the normal control group, each group was treated with ConA (5 mg/l) according to previous researches [22, 23]. The T cells were incubated for 24, 48, and 72 h at 37°C and 5% CO_2_. At each time point, 20 *μ*l of MTT (5 mg/ml) was added to each well (96-well plate), and the cells were further incubated for 4 h at 37°C. Then the supernatants were removed after the 96-well plate was centrifuged at 1800 rpm for 10 min. Then 150 *μ*l DMSO was added to each well to resuspend the T cells after the supernatants were removed. Finally, the optical density at 570 nm was determined by a microplate reader. And the results were from four independent experiments.

### 2.10. Determination of Cytokine Concentrations

The T cells (2 × 10^6^/ml) were divided in the four groups: normal control group (no treatment), ConA group (ConA + T cell), 15 *μ*Μ L-THP group (ConA + T cell + L-THP 15 *μ*Μ), and 30 *μ*Μ L-THP group (ConA + T cell + L-THP 30 *μ*Μ). Each group was treated with ConA (5 mg/L) except for the normal control group. The T cells were incubated for 24, 48, and 72 h at 37°C and 5% CO_2_. At each time point, the supernatants were collected. Finally, the concentrations of TNF-*α* and IL-6 in the supernatants were measured by ELISA according to the manufacturer's instructions. And the results were from four independent experiments.

### 2.11. Statistical Analysis

Data were expressed as the mean ± SD. The results of ELISA, AST, and ALT concentration assays, histopathology, qPCR, immunohistochemistry, Western Blot, and MTT assays were analyzed using Student's *t*-test. All figures were created using GraphPad Prism (v6.0). In all comparisons, *P* values of < 0.05 were considered statistically significant.

## 3. Results

### 3.1. L-THP and 1% DMSO Do Not Affect Normal Mouse Livers

**Figure 1:**
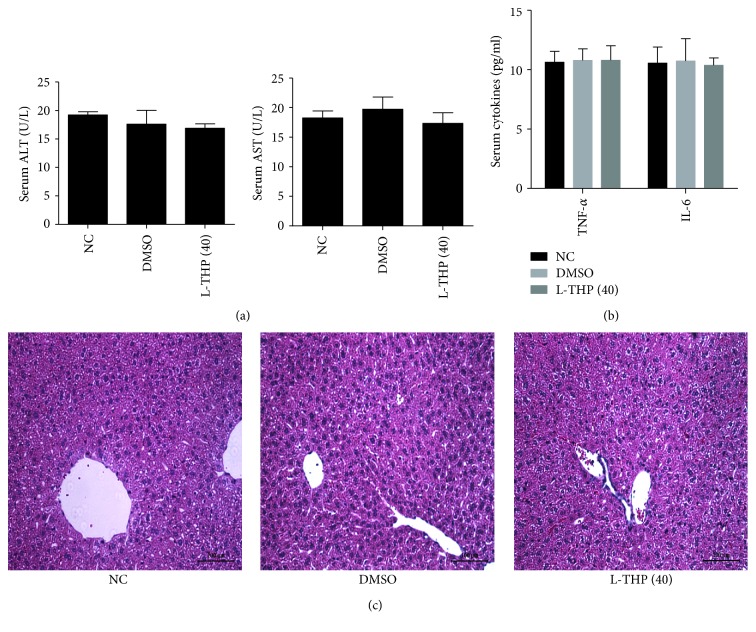
Effects of 1% DMSO and L-THP (40 mg/kg) on the liver function and pathology of mice. (a) The levels of serum ALT and AST in the three groups did not differ. Data were given as means ± SD (*n* = 18, *P* > 0.05). (b) Serum levels of TNF-*α* and IL-6 were measured in the three groups, and the results were expressed as the mean ± SD (*n* = 18, *P* > 0.05). (c) Representative hematoxylin and eosin-stained sections of the liver. Original magnification: ×200.

To determine whether L-THP or 1% DMSO have effects on liver function, we examined the levels of liver enzymes and the release of cytokines in the L-THP and DMSO groups. As shown in Figure 1(a), serum AST and ALT levels did not differ between the 1% DMSO or L-THP groups compared with the control group. Serum TNF-*α* and IL-6 levels were also consistent among all three groups (Figure 1(b)). Additionally, no obvious necrosis was detected by H&E staining (Figure 1(c)).

### 3.2. L-THP Pretreatment Reduced ConA-Induced Liver Injury

**Figure 2:**
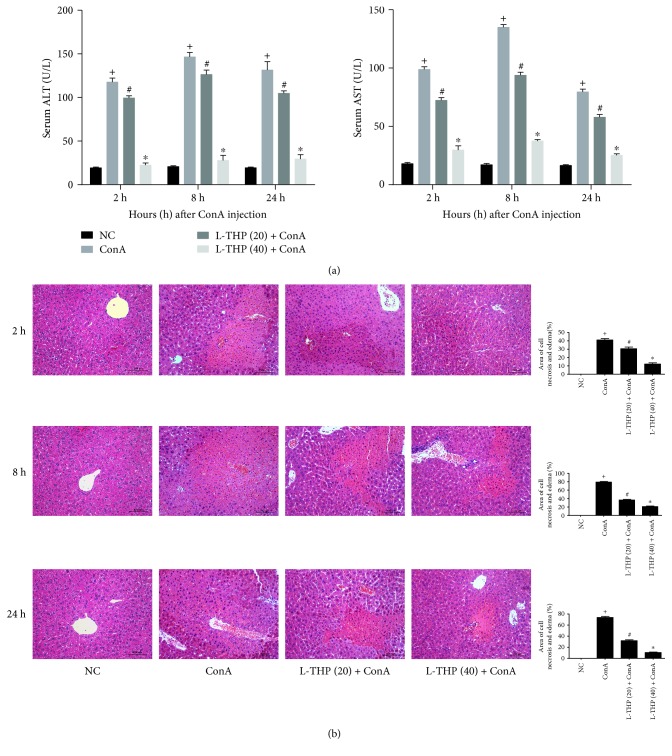
Effects of L-THP on liver function and pathology of mice with ConA-induced acute hepatitis. (a) The levels of serum ALT and AST changed depending on the L-THP dose, 20 mg/kg or 40 mg/kg. Data were given as means ± SD (*n* = 6, ^+^*P* < 0.05 for NC versus ConA, ^#^*P* < 0.05 for L-THP (20) + ConA versus ConA, and ^∗^*P* < 0.05 for L-THP (40) + ConA versus ConA). (b) The necrotic and edematous areas stained with hematoxylin and eosin and used for the liver sections were analyzed with Image-Pro Plus 6.0 (magnification: ×200). The results showed statistically significant differences among the different groups. Data were given as means ± SD (*n* = 6, ^+^*P* < 0.05 for NC versus ConA, ^#^*P* < 0.05 for L-THP (20) + ConA versus ConA, and ^∗^*P* < 0.05 for L-THP (40) + ConA versus ConA).

ConA can activate T cells to rapidly induce AIH. To determine the functions of L-THP on ConA-induced hepatitis, serum and liver tissues were collected 2, 8, and 24 h post-ConA treatment. As shown in Figure 2(a), serum AST and ALT levels were significantly increased at all three time points after the ConA administration. However, L-THP pretreatment reduced the levels of these enzymes in a dose-dependent manner. The histopathological analysis showed the same results. As shown in Figure 2(b), massive necrotic areas were observed in the ConA group. In contrast, the L-THP groups showed minor liver injury at the three time points, indicating that pretreatment with L-THP significantly reduced liver necrosis. Thus, minor liver injury was observed in the high dose group than the low dose group.

### 3.3. L-THP Pretreatment Inhibited the Production of TNF-*α* and IL-6 in ConA-Induced Liver Injury

**Figure 3:**
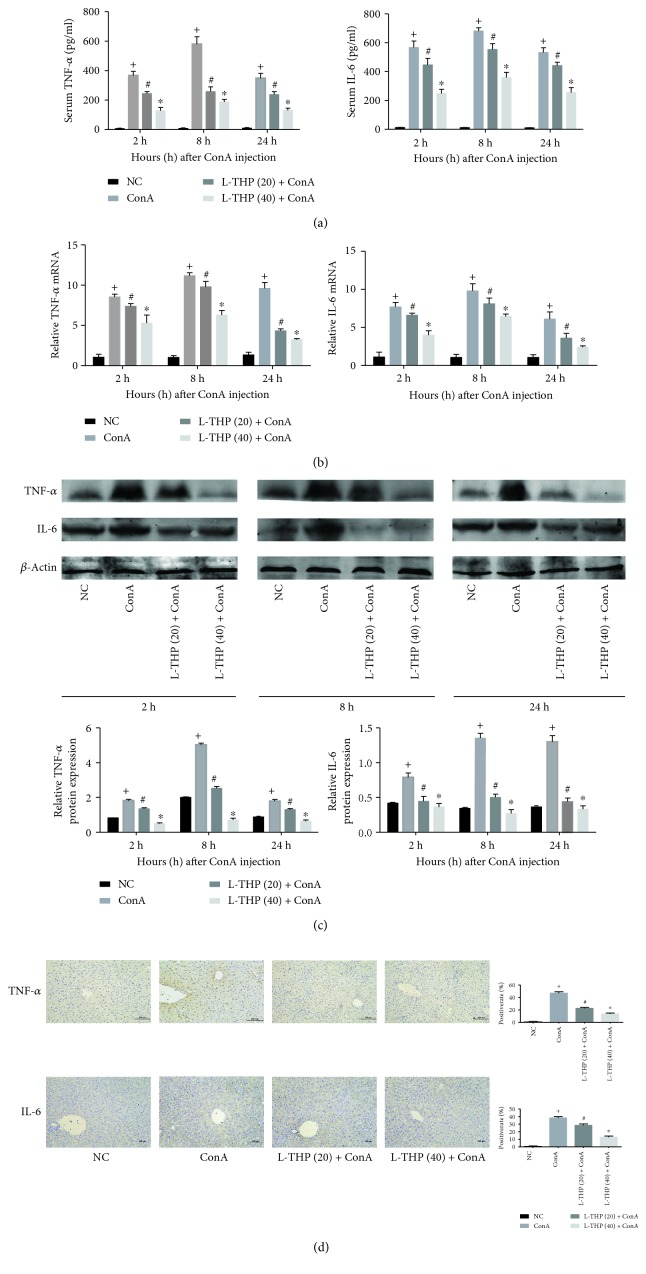
Effects of PSS on the inflammatory response in ConA-induced liver injury in mice. (a) The levels of serum TNF-*α* and IL-6 measured with ELISA were reduced by L-THP pretreatment in mice at doses of 20 mg/kg and 40 mg/kg. Data are presented as means ± SD (*n* = 6, ^+^*P* < 0.05 for NC versus ConA, ^#^*P* < 0.05 for L-THP (20) + ConA versus ConA, and ^∗^*P* < 0.05 for L-THP (40) + ConA versus ConA). (b) qRT-PCR was used to detect the mRNA expression of TNF-*α* and IL-6 in each group, and the results were expressed as the mean ± SD (*n* = 6, ^+^*P* < 0.05 for NC versus ConA, ^#^*P* < 0.05 for L-THP (20) + ConA versus ConA, and ^∗^*P* < 0.05 for L-THP (40) + ConA versus ConA). (c) Western Blot analysis was used to detect the protein expression of TNF-*α* and IL-6. Data were expressed as the mean ± SD (*n* = 6, ^+^*P* < 0.05 for NC versus ConA, ^#^*P* < 0.05 for L-THP (20) + ConA versus ConA, ^∗^*P* < 0.05 for L-THP (40) + ConA versus ConA). (d) The expression of TNF-*α* and IL-6 in liver tissue at 8 h was detected by immunohistochemical staining (Original magnification, ×200) (*n* = 6, ^+^*P* < 0.05 for NC versus ConA, ^#^*P* < 0.05 for L-THP (20) + ConA versus ConA, and ^∗^*P* < 0.05 for L-THP (40) + ConA versus ConA).

Researchers have found that inflammatory response is closely related to the progression of liver injury. As shown in Figure 3(a), the levels of inflammation factors TNF-*α* and IL-6 were determined by ELISA, and the results showed dramatically increased levels of both cytokines in the ConA group. And we used qPCR to detect mRNA levels in tissue from the different groups to verify the results of ELISA. As shown in Figure 3(b), TNF-*α* and IL-6 mRNA expressions increased dramatically in the ConA group, while L-THP pretreatment decreased the mRNA levels of these cytokines in a dose-dependent manner. Additionally, the results of Western Blotting and immunohistochemical staining all indicated that L-THP reduced the inflammatory response in a dose-dependent manner (Figures 3(c) and 3(d)). These results strongly indicated that L-THP pretreatment inhibited the production of inflammatory cytokines in ConA-induced AIH, particularly by the high dose.

### 3.4. L-THP Pretreatment Inhibited Hepatocyte Apoptosis and Autophagy in ConA-Induced Liver Injury

**Figure 4:**
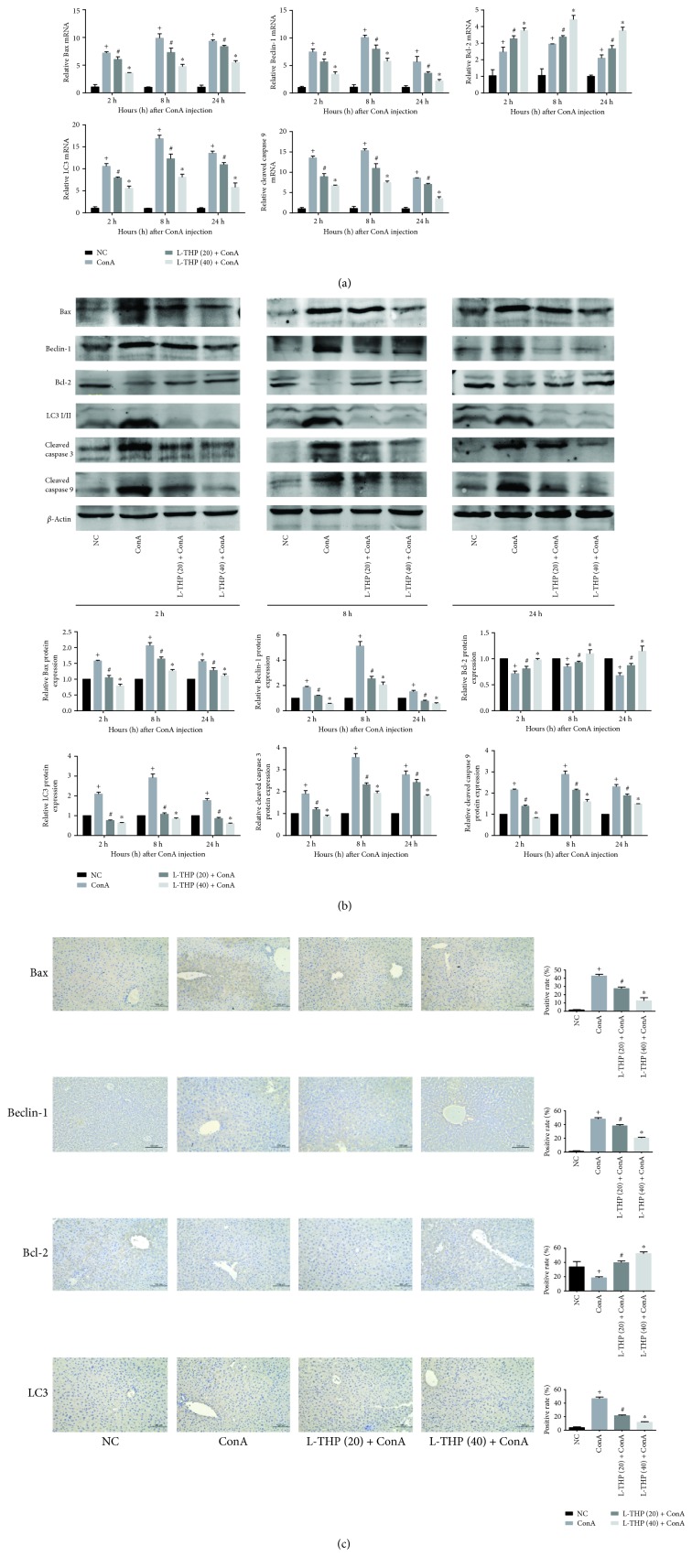
Effects of L-THP pretreatment on apoptosis and autophagy on ConA-induced liver injury in mice. (a) The mRNA expression of Bax, Bcl-2, caspase 9, Beclin-1, and LC3 was detected by qRT-PCR. Data were expressed as the mean ± SD (*n* = 6, ^+^*P* < 0.05 for NC versus ConA, ^#^*P* < 0.05 for L-THP (20) + ConA versus ConA, and ^∗^*P* < 0.05 for L-THP (40) + ConA versus ConA). (b) The protein expression of Bax, Bcl-2, caspase 3, caspase 9, Beclin-1, and LC3 was detected by Western Blotting. Data were expressed as the mean ± SD (*n* = 6, ^+^*P* < 0.05 for NC versus ConA, ^#^*P* < 0.05 for L-THP (20) + ConA versus ConA, and ^∗^*P* < 0.05 for L-THP (40) + ConA versus ConA). (c) Immunohistochemistry staining (×200) showed the expression of Bax, Bcl-2, Beclin-1, and LC3 proteins in liver tissues at 8 h. The positive areas and total area were measured using Image-Pro Plus. Data were expressed as the mean ± SD (*n* = 6, ^+^*P* < 0.05 for NC versus ConA, ^#^*P* < 0.05 for L-THP (20) + ConA versus ConA, and ^∗^*P* < 0.05 for L-THP (40) + ConA versus ConA).

The mRNA and protein expressions of Bcl-2, Bax, Beclin-1, LC3, caspase 3, and caspase 9 were detected by qPCR and Western Blotting, respectively. Bcl-2 is considered an antiapoptosis protein while Bax, caspase 3, and caspase 9 represent the proapoptosis proteins. Beclin-1 and LC3 are commonly considered the markers of autophagy. We applied qPCR and Western Blotting technologies to detect the mRNA and protein expressions of these protein markers, respectively. As shown in Figures 4(a) and 4(b), the expression of Bcl-2 was downregulated by the ConA injection and upregulated by L-THP pretreatment. Conversely, the proapoptotic markers, Bax, caspase 3, and caspase 9, were upregulated by the ConA injection and downregulated by the L-THP pretreatment. LC3 and Beclin-1 showed the highest expression levels in the ConA treatment group, while their expression levels decreased with increasing L-THP doses. The immunohistochemical changes were consistent with the qPCR and Western Blotting results (Figure 4(c)). In conclusion, these results provided strong evidence that L-THP pretreatment attenuated hepatocyte apoptosis and autophagy and reduced pathological damage to the liver following ConA administration.

### 3.5. L-THP Inhibited the TRAF6/JNK Signaling Pathway in ConA-Induced Liver Injury

**Figure 5:**
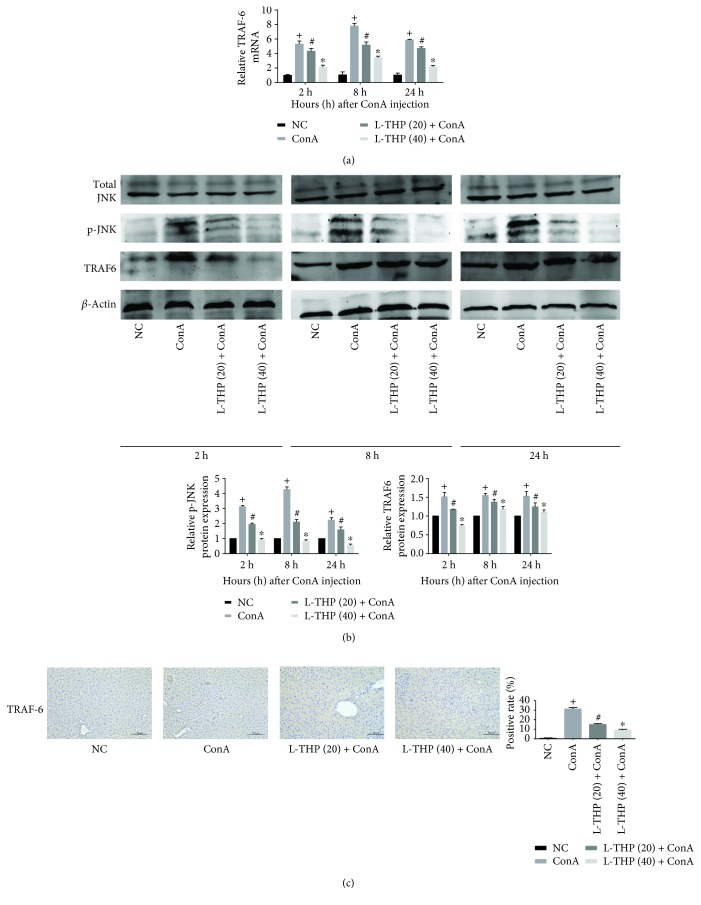
Effects of L-THP on the regulation of the TRAF6/JNK pathway in mice with ConA-induced acute hepatitis. (a) The mRNA level of TRAF6 was detected by qRT-PCR. Data were expressed as the mean ± SD (*n* = 6, ^+^*P* < 0.05 for NC versus ConA, ^#^*P* < 0.05 for L-THP (20) + ConA versus ConA, and ^∗^*P* < 0.05 for L-THP (40) + ConA versus ConA). (b) The expression of TRAF6, total JNK, and p-JNK was determined by Western Blotting. Data were expressed as the mean ± SD (*n* = 6, ^+^*P* < 0.05 for NC versus ConA, ^#^*P* < 0.05 for L-THP (20) + ConA versus ConA, and ^∗^*P* < 0.05 for L-THP (40) + ConA versus ConA). (c) Immunohistochemistry was used to measure the expression of TRAF6 in liver tissues 8 h after ConA injection (original magnification: ×200). Data were expressed as the mean ± SD (*n* = 6, ^+^*P* < 0.05 for NC versus ConA, ^#^*P* < 0.05 for L-THP (20) + ConA versus ConA, and ^∗^*P* < 0.05 for L-THP (40) + ConA versus ConA).

Our data showed that L-THP could attenuate ConA-induced AIH by inhibiting the release of proinflammatory cytokines, such as TNF-*α* and IL-6; however, the underlying mechanism of this activity remained unclear. The TRAF6/JNK pathway is closely related to apoptosis and autophagy. To explore the mechanistic pathway of L-THP in AIH, we measured mRNA and protein levels by qPCR and Western Blotting, respectively (Figures 5(a) and 5(b)). Levels of total JNK protein did not differ between the four groups. However, the levels of phosphorylated JNK and TRAF6 were significantly increased in the ConA-treated group and clearly decreased in the L-THP-pretreated groups at all the three time points. And similar results were found in the immunohistochemistry (Figure 5(c)). These results shown above suggested that L-THP pretreatment could attenuate ConA-induced liver damage via the TRAF6/JNK pathway.

### 3.6. L-THP Suppressed ConA-Induced T Lymphocyte Proliferation *In Vitro*

**Figure 6:**
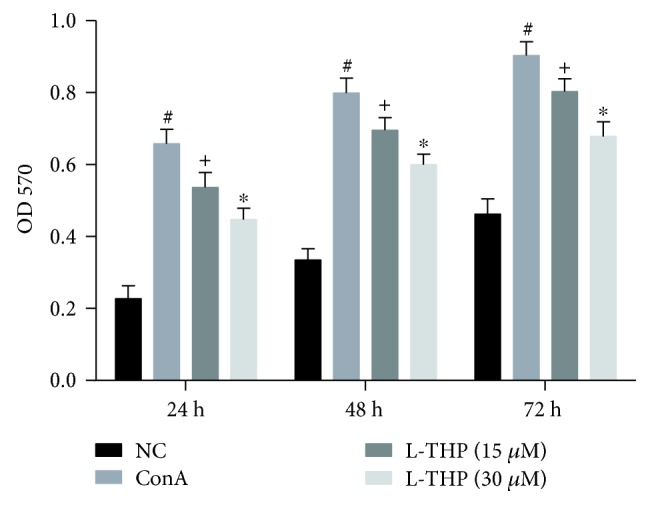
Effects of L-THP on the T lymphocyte proliferation induced by ConA. The proliferation of T lymphocyte treated with ConA and L-THP (15 and 30 *μ*M) was detected with the MTT assay. Data were expressed as the mean ± SD (*n* = 4, ^#^*P* < 0.05 for NC versus ConA, ^+^*P* < 0.05 for L-THP (15 *μ*M) versus ConA, and ^∗^*P* < 0.05 for L-THP (15 *μ*M) versus L-THP (30 *μ*M)).

The MTT assay is commonly used to detect the cell proliferation. As shown in Figure 6, L-THP (15 and 30 *μ*M) suppressed the T lymphocyte proliferation induced by ConA at 24, 48, and 72 h. And L-THP exerted this effect in a dose-dependent manner.

### 3.7. L-THP Reduced the Production of TNF-*α* and IL-6 Induced by ConA *In Vitro*

**Figure 7:**
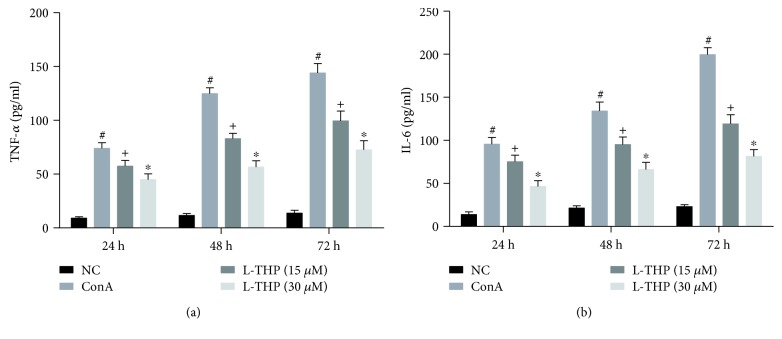
Effects of L-THP on the production of TNF-*α* and IL-6 induced by ConA *in vitro*. The levels of TNF-*α* and IL-6 in the supernatants were detected by ELISA. Data were expressed as the mean ± SD (*n* = 4, ^#^*P* < 0.05 for NC versus ConA, ^+^*P* < 0.05 for L-THP (15 *μ*M) versus ConA, and ^∗^*P* < 0.05 for L-THP (15 *μ*M) versus L-THP (30 *μ*M)).

After incubation for 24, 48, and 72 h at 37°C and 5% CO_2_, the T cell supernatants were collected. And the levels of TNF-*α* and IL-6 in the supernatants were determined by ELISA. As shown in Figure 7, the levels of TNF-*α* and IL-6 increased dramatically in the ConA group compared with the NC group. L-THP (15 and 30 *μ*M) reduced the production of TNF-*α* and IL-6 induced by ConA, and the expression levels of these inflammatory factors in the L-THP (30 *μ*M) group were lower than that in the L-THP (15 *μ*M) group.

## 4. Discussion

AIH is considered a rare immunological liver disease; however, the incidence of AIH has been increasing in recent years, resulting in a significant global health burden. Corticosteroids, alone or in combination with azathioprine, have been used in the clinic as the standard treatment for AIH for many years. However, researches have indicated that up to 20% of patients do not respond or are intolerant to this treatment [24]. Moreover, side effects, such as impaired immunity and a disturbed endocrine function, have been observed in many patients receiving this standard treatment [25]. Therefore, new effective therapeutic options for AIH are urgently needed.

The ConA model is easy to use and is regarded as the best experimental model for AIH research. Inflammatory cytokines such as TNF-*α* and IL-6 play essential roles in the pathogenesis of ConA-induced liver injury [3, 26]. L-THP had anti-inflammatory effect by downregulating the expression of the inflammatory cytokines TNF-*α* and IL-6 [19, 27, 28]. However, function of L-THP pretreatment in ConA-induced AIH remains unknown.

In our study, we investigated the mechanism underlying the anti-inflammatory activity of L-THP in the ConA-induced AIH model. The decreases in serum levels of liver enzymes, range of necrosis of liver tissues, and release of proinflammatory cytokines indicated that L-THP ameliorated ConA-induced hepatitis. ALT and AST are both transaminases widely used as markers to determine the extent of liver injury [9, 29]. The levels of serum ALT and AST were elevated in the ConA group, whereas they were reduced markedly by both doses of L-THP (20 and 40 mg/kg), particularly by the high dose group, and the changes in pathological damage showed the same results. Furthermore, results of qPCR and Western Blot indicated that the release of TNF-*α* and IL-6 could be suppressed by the L-THP pretreatment. And results of the MTT assay and ELISA showed that L-THP (15 and 30 *μ*M) could suppress T lymphocyte proliferation and the production of TNF-*α* and IL-6 induced by ConA in a dose-dependent manner *in vitro*. We considered whether L-THP could exhibit hepatoprotective functions by inhibiting the interaction between TNF-*α* and its receptor, tumor necrosis factor receptor (TNFR).

Tumor necrosis factor receptor–associated factor (TRAF) proteins play important roles in signaling through a variety of adaptive and innate immune receptors as well as cytokine receptors [30]. TRAF6 is expressed in mammalian tissues and can activate multiple signaling pathways, including the JNK pathway. Previous studies have shown that the TRAF6 expression is closely related to many diseases, such as cancer [31, 32] and autoimmune diseases [33]. Moreover, several studies have shown that liver damage could be effectively attenuated by inhibiting the TRAF6 pathway [34, 35]. This study showed that TRAF6 expression was increased following ConA injection but was inhibited by L-THP, particularly by high doses. C-Jun N-terminal kinase (JNK) is an important member of the mitogen-activated protein kinase (MAPK) cascade that can be activated by many stimuli, such as immune responses, cell stress, hormones, drugs, and inflammatory cytokines. JNK plays a key role in TNF-*α*-induced apoptosis [36, 37]. p-JNK, the active form of JNK, is also closely associated with ConA-induced liver injury. p-JNK was generated after ConA injection and translocated to the nuclear or mitochondrial membrane, causing liver damage. Li et al. found that the antioxidant astaxanthin could exert protective effects on ConA-induced AIH by inhibiting JNK phosphorylation *in vivo* and *in vitro* [38]. The recruitment of TRAF6 to inflammatory receptors plays a key role in JNK activation [39]. Lu et al. found in the model of spinal cord atrocities, TNF-*α* and JNK pathways could be integrated by upregulating TRAF6 to cause neuropathic pain [40]. In this study, Western Blotting and immunohistochemistry demonstrated high levels of p-JNK and TRAF6 in the ConA group but low levels in the L-THP-pretreated groups. These results indicated that L-THP reduced TRAF6 expression by inhibiting TNF-*α* release, which subsequently inhibited JNK phosphorylation.

Previous studies have shown that apoptosis is essential for inflammatory-related liver disease [10, 41, 42]. To investigate how L-THP reduced liver injury and modulated JNK phosphorylation, we detected the levels of liver cell apoptosis. Apoptosis or programmed cell death can be induced by endogenous or exogenous factors. Bcl-2 is an antiapoptotic protein, while Bax is a proapoptotic protein; the Bcl-2 : Bax ratio determines cell survival or apoptosis [43]. Studies have shown that JNK can translocate to the mitochondrial membrane where it phosphorylates (inactivates) Bcl-2, leading to the release of cytochrome C to initiate caspase-mediated apoptosis [44–46]. In this study, PCR and Western Blotting indicated that L-THP pretreatment upregulated Bcl-2 and downregulated Bax, caspase 3, and caspase 9. These results demonstrated that L-THP attenuated hepatic cell apoptosis in ConA-induced hepatitis by inhibiting JNK phosphorylation.

**Figure 8:**
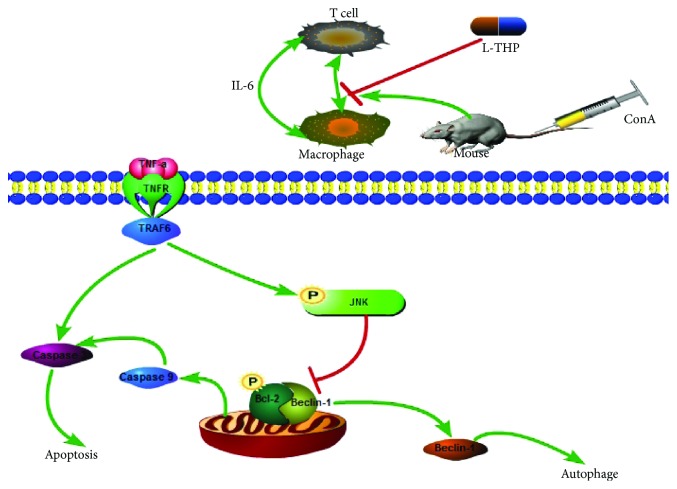
Mechanism of the L-THP action. In ConA-induced autoimmune hepatitis, L-THP reduces autophagy by inhibiting the JNK/p-JNK pathway. After ConA injection, TNF-*α* combined with TNFR and TRAF6, which was expressed on the surface of hepatocytes. This led to the activation of JNK, which phosphorylated Bcl-2, then promoting the release of caspase 9 and caspase 3, causing apoptosis. Inactive Bcl-2 dissociated from Beclin-1, enhancing the induction of autophagy. Taking these all together, L-THP successfully inhibited the release of TNF-*α* and ameliorated hepatocyte apoptosis and autophagy by reducing the phosphorylation of JNK.

Autophagy, or type II programmed cell death, is a catabolic process that is associated with maintaining cellular homeostasis [47]. Studies have shown that autophagy plays an essential negative regulatory role in the liver. Beclin-1 and LC3 are markers of autophagy. Recently, Bcl-2 was shown to play an important role in the crosstalk between apoptosis and autophagy by interacting with Beclin-1 [48]. JNK phosphorylation inactivates Bcl-2, causing the displacement of Bcl-2 from Beclin-1, allowing free Beclin-1 to induce autophagy (Figure 8). The transformation of LC3I to LC3II is closely associated with the formation of autophagosomes. In our study, the results of qPCR and Western Blotting indicated that Beclin-1 and LC3 expressions were reduced by L-THP pretreatment. These results indicated that L-THP could reduce ConA-induced liver damage by inhibiting apoptosis and autophagy.

The mechanisms underlying the protective role of L-THP in liver injury are complex and require more investigation. While we began this line of investigation in this study, these experiments had several limitations. We suggest that the pathogenesis of AIH should be further investigated to facilitate research into the molecular markers, AIH diagnostics, and targeted therapies [3–5]. Moreover, we hope that more studies focused on the molecular mechanisms of hepatic inflammation, injury, and repair will be conducted in the future. For example, the signaling pathway(s) and pathways that promote inflammation and fibrosis [49, 50], inflammatory-related liver disease [10, 41, 51, 52], and inflammation and cancer [53, 54] should be further explored to achieve early treatment and/or delay disease progression.

## 5. Conclusions

This study demonstrated that L-THP suppressed ConA-induced liver injury in mice. Furthermore, L-THP reduced the immunoreactions and pathological damage associated with this model by inhibiting inflammatory factors such as TNF-*α* and IL-6. L-THP could also suppress T lymphocyte proliferation and the production of TNF-*α* and IL-6 induced by ConA *in vitro*. The antiapoptotic and antiautophagy effects of L-THP depended on inhibiting TNF-*α*-mediated TRAF6/JNK signaling. Taken together, these findings suggest that L-THP may be a potential therapeutic agent for AIH.

## Figures and Tables

**Table 1:** Sequences of primers used for qPCR.

Gene	DNA strand	Primer sequence (5′-3′)
*β*-Actin	Forward	GGCTGTATTCCCCTCCATCG
	Reverse	CCAGTTGGTAACAATGCCATGT

TNF-*α*	Forward	CAGGCGGTGCCTATGTCTC
	Reverse	CGATCACCCCGAAGTTCAGTAG

IL-6	Forward	CTGCAAGAGACTTCCATCCAG
	Reverse	AGTGGTATAGACAGGTCTGTTGG

Bax	Forward	AGACAGGGGCCTTTTTGCTAC
	Reverse	AATTCGCCGGAGACACTCG

Bcl-2	Forward	GCTACCGTCGTCGTGACTTCGC
	Reverse	CCCCACCGAACTCAAAGAAGG

Beclin-1	Forward	ATGGAGGGGTCTAAGGCGTC
	Reverse	TGGGCTGTGGTAAGTAATGGA

LC3	Forward	GACCGCTGTAAGGAGGTGC
	Reverse	AGAAGCCGAAGGTTTCTTGGG

TRAF6	Forward	TCATTATGATCTGGACTGCCCAAC
	Reverse	TGCAAGTGTCGTGCCAAGTG

## Data Availability

The data used to support the findings of this study are available from the corresponding author upon request.
